# Synthesis, Characteristics, and Field Applications of High-Temperature and Salt-Resistant Polymer Gel Tackifier

**DOI:** 10.3390/gels11060378

**Published:** 2025-05-22

**Authors:** Guowei Zhou, Xin Zhang, Weijun Yan, Zhengsong Qiu

**Affiliations:** 1 CNPC Greatwall Drilling Company, 101 Anli Road, Chaoyang District, Beijing 100101, China; zhgw1027@163.com (G.Z.); zxin.gwdc@cnpc.com.cn (X.Z.); 13792869249@163.com (W.Y.); 2 Petroleum Engineering Institute, Qingdao Campus, China University of Petroleum (East China), No.66 Changjiang East Road, Huangdao District, Qingdao 266000, China

**Keywords:** polymer gel, high temperature resistance, calcium carbonate, nano, salt resistance

## Abstract

To address the technical challenge of high polymer gel viscosity reducers losing viscosity at elevated temperatures and difficulty in controlling fluid loss, a polymer-based nano calcium carbonate composite high-temperature tackifier named GW-VIS was prepared using acrylamide (AM), 2-acrylamido-2-methylpropanesulfonic acid (AMPS), N-vinylpyrrolidone (NVP), and nano calcium carbonate as raw materials through water suspension polymerization. This polymer gel can absorb water well at room temperature and has a small solubility. After a long period of high-temperature treatment, most of it can dissolve in water, increasing the viscosity of the suspension. The structure of the samples was characterized by infrared spectroscopy, thermogravimetric analysis, and scanning electron microscopy, and their performance was evaluated. Rheological tests indicated that the 0.5% water suspension had a consistency coefficient (k = 761) significantly higher than the requirement for clay-free drilling fluids (k > 200). In thermal resistance experiments, the material maintained stable viscosity at 180 °C (reduction rate of 0%), and only decreased by 14.8% at 200 °C. Salt tolerance tests found that the viscosity reduction after hot rolling at 200 °C was only 17.31% when the NaCl concentration reached saturation. Field trials in three wells in the Liaohe oilfield verified that the clay-free drilling fluid supported formation operations successfully. The study shows that the polymer gel has the potential to maintain rheological stability at high temperatures by forming a network structure through polymer chain adsorption and entanglement, with a maximum temperature resistance of up to 200 °C, providing an efficient drilling fluid for deep oil and gas well development. It is feasible to select nano calcium carbonate to participate in the research of high-temperature resistant polymer materials. Meanwhile, the combined effect of monomers with large steric hindrance and inorganic materials can enhance the product’s temperature resistance and resistance to NaCl pollution.

## 1. Introduction

The deeply buried fractured formations in the Bohai Bay region of China are widely distributed, with significant exploration and development potential [1]. Over the years, CNOOC and CNPC have conducted extensive development in this area, including many high-temperature exploration wells with downhole temperatures generally above 150 °C. The formations for these exploration wells are deep-buried hill reservoirs, which are deeply buried with high temperatures and many fractures. Some oil layers have temperatures as high as 200 °C, but the formation pressure coefficients are only 1.01–1.06, representing a high-temperature and low-pressure oil and gas reservoir [2]. The oil and gas channels in these formations are mainly micro-fractures, making them particularly prone to oil and gas layer contamination. Therefore, it is necessary to select clay-free water-based drilling fluids with good reservoir protection effects during drilling [3].

In the clay-free drilling fluid system, the most crucial treatment agent is the tackifier. Water-based drilling fluid tackifiers have been developed for many years, with existing products mainly researched and developed from four aspects: natural plant gum tackifiers, inorganic material tackifiers, organic polysaccharide tackifiers, and synthetic polymer tackifiers [4]. Natural plant gums include guar gum, alginate, etc., which are obtained through physical processing of endosperm in plant seeds, including guar gum, locust bean gum, fenugreek gum, etc. Inorganic material tackifiers include layered metal hydroxides, mixed metal silicates, etc. Organic polysaccharide tackifiers include polyanionic cellulose, hydroxyethyl cellulose, xanthan gum, etc. Synthetic polymers include acrylamide-based polymers, acrylic acid-based polymers, etc. [5,6,7,8,9]

Most high-temperature-resistant water-based drilling fluid tackifiers are synthetic polymer materials, prepared by polymerizing monomers with high-temperature resistance. Hu prepared a low-solid-phase drilling fluid tackifier using acrylamide and acrylic acid as raw materials, obtaining the optimal ratio and synthesis conditions through orthogonal experiments, and the product has good viscosity-enhancing performance [10]. Peiffer prepared an amphoteric polymer tackifier using 2-acrylamido-2-methylpropanesulfonic acid (AMPS), N-vinylpyrrolidone (NVP), and sodium styrene sulfonate as raw materials. Due to its amphoteric nature, it has strong salt resistance and better viscosity in brine than conventional ionic and non-ionic tackifiers. Yan developed an inorganic cationic tackifier, which can significantly improve the plastic viscosity of drilling fluids, and was applied in two wells in Shengli Oilfield, showing significant viscosity-enhancing performance and good salt resistance, ensuring the smooth construction of oil and gas wells [11]. Pu developed a soil-free drilling fluid tackifier resistant to 160 °C indoors, which also had certain reservoir protection effects [12]. Lu prepared a high-temperature-resistant tackifier using monomers containing sulfonic acid and cationic groups as raw materials [13]. When used in high-temperature resistant clay-free drilling fluids, it can improve the temperature resistance and viscosity-enhancing ability of the drilling fluid and also has certain reservoir protection effects. Tian using AMPS and dimethyldiallylammonium chloride as raw materials, developed a high-temperature resistant polymer tackifier through water suspension polymerization. The product can increase the apparent viscosity of drilling fluids by three times, showing good viscosity-enhancing effects [14]. Since 1988, McCormick has focused on molecular structure, understanding it from different perspectives, and analyzing hydrophobic associations in depth. They discussed the performance of polymers from the perspectives of viscosity, molecular weight, and molecular chain structure [15]. In suspensions, the molecular interaction force generated by polyelectrolyte complexes is very large. In this case, the freedom of polymer chains in the components decreases, the hydrodynamic volume between macromolecules increases, and the hydrophobic association generated within polymer molecules can also enhance the viscosity of polymers under certain conditions. Candau fully integrated reverse emulsion with microemulsion and used various methods to study amide polymers [16]. The US patent US7098171 introduced a new type of polymer, a high-temperature and salt-resistant fluid loss control tackifier, which contains AM, AMPS, and other chemical substances in its monomers. Its temperature resistance is very significant, and the synthetic preparation method is produced by free radical copolymerization at specific ratios.

In the realm of drilling fluid technology, there exists a demand for multifunctional products. The incorporation of nano calcium carbonate as a raw material enables the formulated product to demonstrate not only viscosity-enhancing properties but also reduced fluid loss within the drilling fluid. This dual functionality forms the rationale for selecting nano calcium carbonate as a constituent in our synthetic formulation.

The selection of acrylamide (AM), 2-acrylamido-2-methylpropane sulfonic acid (AMPS), and N-vinylpyrrolidone (NVP) as co-monomers is based on their distinct molecular advantages. AM and AMPS provide strong adsorption functional groups, while polymeric products derived from NVP exhibit significant steric hindrance. These monomer-derived polymers have demonstrated validated thermal stability in downhole conditions [10,11,12,13,14,15,16].

Although extensive studies have documented the application of nano silica in polymer synthesis to enhance thermal resistance and plugging capabilities, our preference for nano calcium carbonate arises from three strategic considerations: first, its cost-effectiveness (approximately one-tenth of nano silica’s price); second, its superior high-temperature resistance with a more stable crystalline structure under thermal stress; and third, its environmentally benign synthesis pathway utilizing CO_2_ as precursor material, thereby contributing to carbon emission reduction initiatives.

Due to the lack of independently developed high-temperature-resistant tackifiers for clay-free water-based drilling fluids, the company could only use products from other companies for drilling in deep wells above 180 °C. The high cost and unreliable supply of these products severely impacted drilling safety and efficiency. Developing a new tackifier was therefore essential.

In summary, this paper develops a new type of high-temperature resistant tackifier through the selection of high-temperature resistant monomers and a combination of inorganic and organic materials. This tackifier can be used for both clay-free drilling fluid and conventional water-based drilling fluid. At the same time, due to its excellent viscosification ability, it is also promising in oil recovery, providing technical support for the development of the company’s high-temperature resistant water-based drilling fluid technology.

## 2. Results and Discussion

### 2.1. Infrared Spectroscopy Analysis of GW-VIS

**Figure 1:**
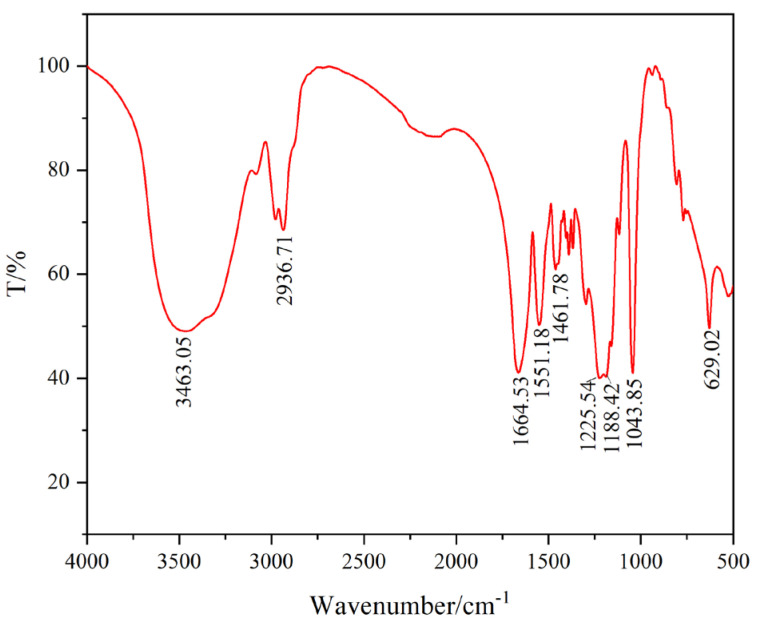
Infrared spectrum curve of GW-VIS.

After washing, purifying, and crushing, we added KBr and GW-VIS for tablet pressing and sample preparation. A Fourier infrared spectrometer was used to test its infrared spectrum, and the results are shown in Figure 1.

From the curve, it can be seen that the characteristic absorption peak at 3463.05 cm^−1^ is due to hydroxyl groups, the characteristic absorption peak at 2936.71 cm^−1^ is due to methyl groups, the characteristic absorption peak at 1551.18 cm^−1^ is due to amide groups, the characteristic absorption peak at 1664.53 cm^−1^ is due to carbonyl groups, the characteristic absorption peak at 1225.54 cm^−1^ is due to C-N bonds, the characteristic peak at 1461.78 cm^−1^ is due to C-O bonds in carbonate groups, and the characteristic absorption peaks at 1043.85 cm^−1^ and 1188.42 cm^−1^ are due to sulfonic acid groups [17,18,19,20,21,22,23]. This indicates that the synthesized product contains hydroxyl, methyl, amide, carbonyl, carbonate, and sulfonic acid groups. It means acrylamide (AM), AMPS, calcium carbonate (CaCO_3_), and NVP all participated in the synthesis of GW-VIS. As a core treatment agent for clay-free water-based drilling fluid, it needs to have good viscosity-enhancing effects. Therefore, the polymer gel GW-VIS is required to contain more adsorption groups, and amide, carbonyl, and sulfonic acid groups all have good adsorption properties, which is conducive to the mutual adsorption and entanglement of tackifier molecules in water, forming a network structure, improving the viscosity and shear force of the clay-free water-based drilling fluid [24].

### 2.2. Thermogravimetric Analysis of GW-VIS

**Figure 2:**
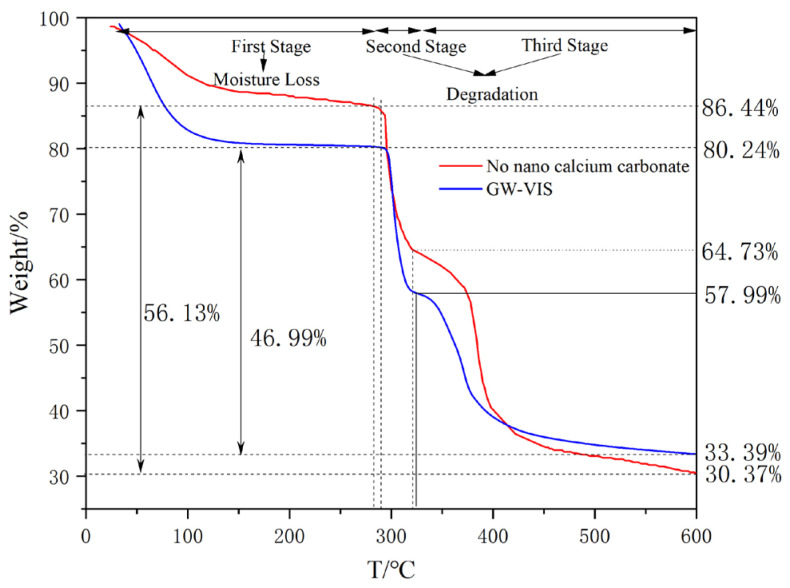
Thermogravimetric curves of GW-VIS and tackifier without nano calcium carbonate.

Under identical heating rates, the initial stage corresponding to moisture elimination demonstrated that the tackifier without nano calcium carbonate exhibited both a lower mass loss rate and reduced total moisture loss compared to GW-VIS, indicating its inferior moisture retention capacity (Figure 2). During the secondary decomposition phase, the degradation temperature of GW-VIS exhibited a 5 °C elevation relative to the tackifier without nano calcium carbonate. Similar thermal behavior patterns were observed in the third stage. Aggregate analysis of stages II and III revealed mass losses of 56.07% and 46.85% for the tackifier without nano calcium carbonate and GW-VIS, respectively. After normalizing for the 1% nano calcium carbonate loading in GW-VIS, this composite demonstrated an 8.22% reduction in total mass loss compared to the tackifier without nano calcium carbonate. This differential thermal degradation profile conclusively establishes the enhanced thermal resistance characteristics imparted to GW-VIS through nano calcium carbonate incorporation.

Based on established theoretical frameworks, this study demonstrates that the interaction between nano calcium carbonate and the polymer matrix in the present work operates through a synergistic combination of physical entanglement and chemical bonding, leading to enhanced overall performance of the resulting composite. As corroborated by the 2020 study conducted by Francis’s team at the University of Wesleyan (USA) in Macromolecules, the presence of strong interfacial interactions between nanoparticles and the polymer matrix induces the formation of a “bound polymer layer” on the nanoparticle surfaces, which significantly promotes thermal stability [25].

In our article, the polymer segments are anchored at the nano-CaCO_3_/polymer interface, effectively restricting the thermal motion of the polymer chains and thereby improving the composite’s thermal resistance. This mechanism is validated by thermogravimetric analysis (TGA) data. The GW-VIS exhibits superior thermal stability compared to the polymer without nano calcium carbonate, as evidenced. Furthermore, Fourier-transform infrared (FTIR) spectroscopy confirms the participation of nano calcium carbonate in chemical interactions.

### 2.3. Scanning Electron Microscopy Analysis of GW-VIS

**Figure 3:**
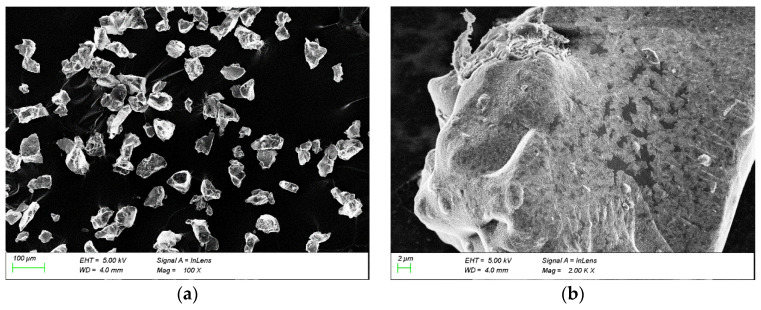
SEM pictures of GW-VIS. (**a**) Enlarge by 100 times (**b**) Enlarge by 2000 times.

The solubility of polymers in water is influenced by several factors, with the geometric shape of the molecular chains being the most significant [26]. Linear and branched polymers are generally easier to dissolve because their molecular structures are more open, facilitating interaction with water molecules. As long as they can swell indefinitely with water, they may dissolve. However, larger molecular weights increase the difficulty and slow down the rate of dissolution [27]. On the other hand, bulk (crosslinked) polymers form a three-dimensional network structure due to numerous crosslinking bonds between molecules, making it difficult for water molecules to penetrate; thus, they typically only swell rather than dissolve. The degree of crosslinking affects the swelling capacity; higher crosslinking reduces swelling, but appropriate control of crosslinking can enhance the thermal resistance of polymers without significantly affecting their solubility [28]. Sigma300 (MIT: Cambridge, MA, USA) scanning electron microscopy was used to observe the microstructure of the polymer gel GW-VIS (Figure 3). The SEM images show that the synthesized product exhibits irregular block shapes with rough surfaces and numerous fine pores and cracks. During the synthesis of GW-VIS, some crosslinkers were added, resulting in a certain degree of crosslinking that affects solubility but can also improve the thermal resistance of the product. Although the high molecular weight of GW-VIS affects its solubility, the SEM images reveal numerous micro-pores and cracks, allowing water molecules to more easily enter the gel, thereby accelerating the gel’s swelling.

### 2.4. Viscosity of GW-VIS Water Suspensions

#### 2.4.1. Viscosity Measured by Six-Speed Rotational Viscometer

The viscosity of water suspensions with concentrations of 0.05%, 0.1%, 0.25%, 0.5%, 0.75%, and 1% GW-VIS was tested using a six-speed rotational viscometer to analyze its viscosity-enhancing performance. The *n*-value and *K*-value of the water suspension were calculated from readings at 600 rpm and 300 rpm to analyze its rock-carrying capacity. The *n*-value, known as the flow index, represents the degree of non-Newtonian behavior of pseudoplastic fluids within a certain range of flow rates; fluids are pseudoplastic when *n* < 1. The *K*-value, known as the consistency coefficient, indicates the viscosity of the fluid; a higher *K*-value means greater viscosity [29]. For drilling fluids, the *n*-value is generally between 0.4 and 0.7.

**Figure 4:**
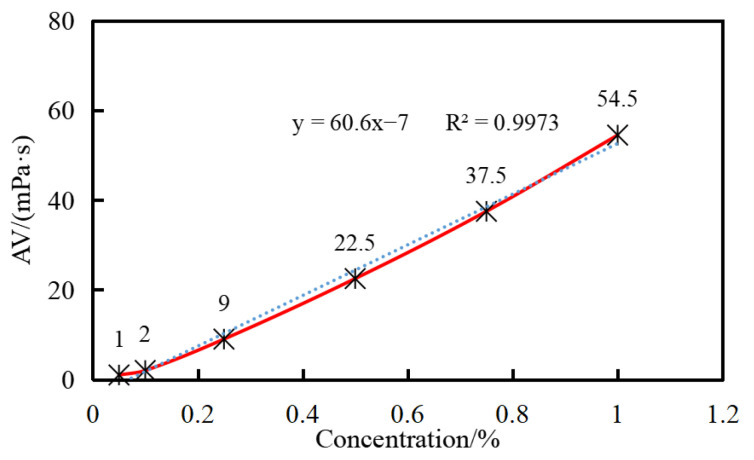
Apparent viscosity of GW-VIS water suspensions—six speed rotational viscometer. (The solid line is a curve formed by connecting data points and the dotted line represents the fitting curves of the apparent viscosity at four concentrations: 0.25%, 0.5%, 0.75% and 1%).

The viscosity of the water suspension increases with the concentration of GW-VIS and shows a good linear relationship (Figure 4). Due to the smaller readings and larger errors at concentrations of 0.05% and 0.1%, data fitting was performed using the viscosity data from the other four concentrations. The fitted data formula shows a variance of 0.9973, indicating a good linear relationship between the viscosity of the suspension and the concentration of GW-VIS. During drilling operations, the required concentration can be deduced from the desired viscosity data according to this formula. For vertical well drilling, the *K*-value of the clay-free drilling fluid should be maintained above 200, and the *K*-value of the 0.5% polymer gel tackifier water suspension can reach 761, which is well above 200, meeting the requirements for drilling operations in relevant formations (Table 1) [30]. Therefore, a recommended dosage of 0.5% is suggested for the clay-free drilling fluid.

#### 2.4.2. Viscosity Measured by Brookfield Viscometer

The viscosity of water suspensions with concentrations of 0.05%, 0.1%, 0.25%, 0.5%, 0.75%, and 1% GW-VIS was tested using a Brookfield viscometer to analyze its viscosity-enhancing performance.

**Figure 5:**
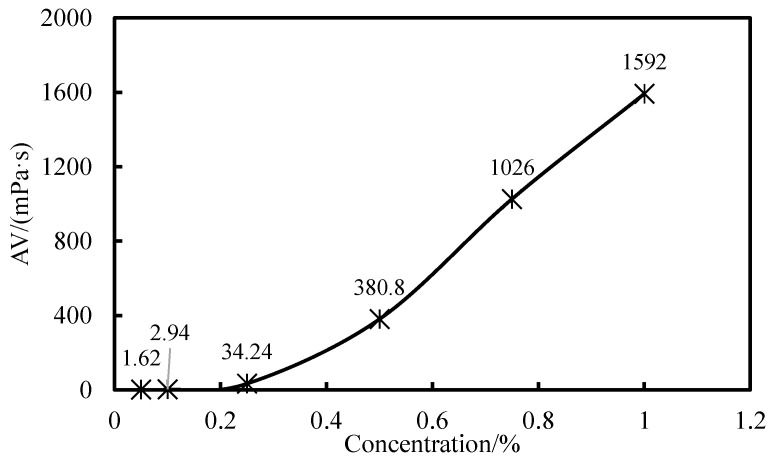
Viscosity of GW-VIS water suspensions—Brookfield viscometer.

**Figure 6:**
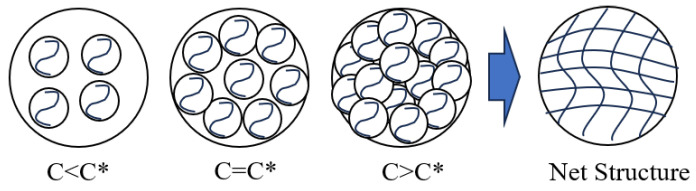
Formation process of polymer suspension network structure (C* is the critical overlap concentration).

The rheological behavior of GW-VIS water suspension shows a significant concentration effect. As shown in the curve (Figure 5), the apparent viscosity increases in a two-stage pattern with the increase in GW-VIS content. When the concentration is ≤0.25%, the suspension maintains a low viscosity. After the concentration exceeds the critical threshold of 0.25%, the viscosity increases linearly with the concentration. This trend is consistent with the multi-shear rate measurement results of the six-speed rotational viscometer (Figure 4). The polymer gel tackifier developed in this paper is a high molecular polymer, and its viscosity increase requires each molecule to fully or partially unfold and entangle in water, forming a network structure. The viscosity follows the three-stage model of the Flory–Huggins suspension theory [31]. In the first stage, the dilute suspension region (C < C*): When the mass fraction is ≤0.25%, the polymer molecules exhibit an isolated globular conformation, with large intermolecular distances, resulting in a near-zero probability of entanglement, and the hydrogen-bonded hydration layer, which mainly contributes to viscosity, cannot form a continuous phase, thus exhibiting Newtonian fluid characteristics [32]. In the second stage, the critical overlap region (C = C*): At the 0.25% concentration point, the probability of molecular contact increases sharply, and the suspension enters the semi-dilute boundary region, where the viscosity shows exponential growth. In the third stage, the concentrated suspension region (C > C*): After exceeding the critical overlap concentration, as shown in Figure 6, the molecular chains form a three-dimensional entangled network, showing typical viscoelastic behavior, and the suspension viscosity increases rapidly.

### 2.5. Thermal Resistance Performance of GW-VIS Water Suspensions

The rheological properties of a 1 wt% polymer gel water suspension were tested before and after hot rolling at 180 °C for 16 h using a six-speed rotational viscometer. Compared to the pure water suspension of GW-VIS in Section 2.3, a 0.5% thermal stabilizer (anhydrous sodium bisulfite) was added when testing the thermal resistance performance of the polymer gel. The apparent viscosity of the water suspension decreased from 54.5 mPa·s to 27.5 mPa·s before hot rolling due to the addition of Na^+^, which inhibited the ionization of Na^+^ in the polymer gel molecules, reducing the intramolecular repulsion and causing the polymer molecular chains to curl, thereby decreasing the viscosity [33]. Even so, the water suspension still had excellent viscosity-enhancing performance at a 1% dosage, with a yield point and plastic viscosity ratio of 0.62 before hot rolling and 0.53 after hot rolling (Table 2), which is still higher than the required value of 0.36 [34]. At the same time, after 16 h of hot rolling at 180 °C, the viscosity of the polymer gel tackifier water suspension did not change, with a viscosity reduction rate of 0. According to the relevant calculation formula, the n-value of the water suspension before hot rolling was 0.51, and the K-value was 844; after hot rolling, the n-value was 0.55, and the K-value was 656. When using a clay-free drilling fluid for vertical well drilling, the K-value should be greater than 200, so the developed tackifier has very good application potential in clay-free drilling fluids.

To analyze its thermal resistance, the polymer gel tackifier water suspension was hot rolled at 180 °C, 200 °C, and 220 °C for 16 h. The viscosity reduction rate after hot rolling at 180 °C was 0%, at 200 °C it was 14.8%, and at 220 °C it was 50.91%. The product has good thermal resistance and can be used for drilling operations in oil and gas wells requiring clay-free drilling fluids with circulating temperatures up to 200 °C.

### 2.6. Performance of GW-VIS in Saltwater

In a 1 wt% GW-VIS water suspension, NaCl concentrations of 0.5 wt%, 1 wt%, 2.5 wt%, and 5 wt% were added, and the suspensions were hot rolled at 180 °C for 16 h in a roller furnace. The apparent viscosity of the polymer gel tackifier water suspension was tested using a six-speed rotational viscometer, and the effect of different concentrations of NaCl on the viscosity of the suspension before and after hot rolling, as well as the viscosity reduction rate of different drilling fluids after hot rolling, was calculated.

**Figure 7:**
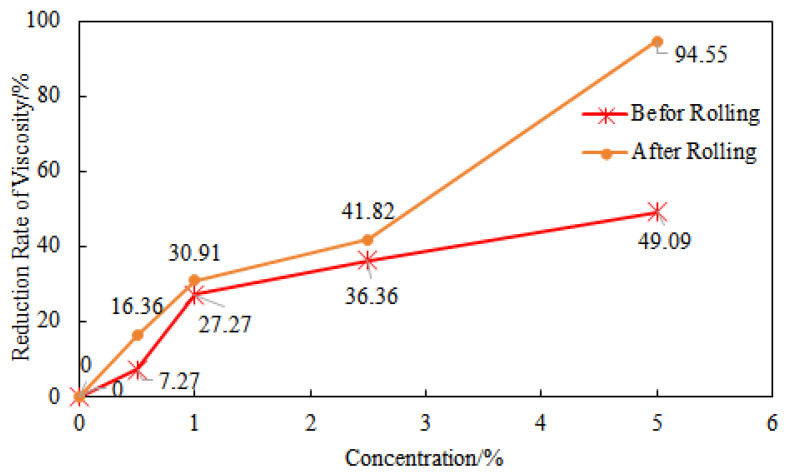
Effect of NaCl on viscosity of drilling fluid before and after hot rolling.

Using the initial drilling fluid without NaCl as a control parameter, the viscosity reduction rate of the drilling fluid gradually increased with the increase in NaCl concentration before and after hot rolling (Figure 7). The curve showing the effect of NaCl on the viscosity of the drilling fluid before hot rolling indicates that when the NaCl concentration is less than 1%, the slope of the viscosity reduction rate curve is 27.27, and after the NaCl concentration exceeds 1%, the slope of the viscosity reduction rate curve changes to 5.455, significantly slowing down the rate of viscosity decrease. The curve showing the effect of NaCl on the viscosity of the drilling fluid after hot rolling also follows the same pattern, but when the NaCl concentration is 5%, the viscosity reduction rate of the drilling fluid after hot rolling increases significantly compared to before hot rolling.

**Figure 8:**
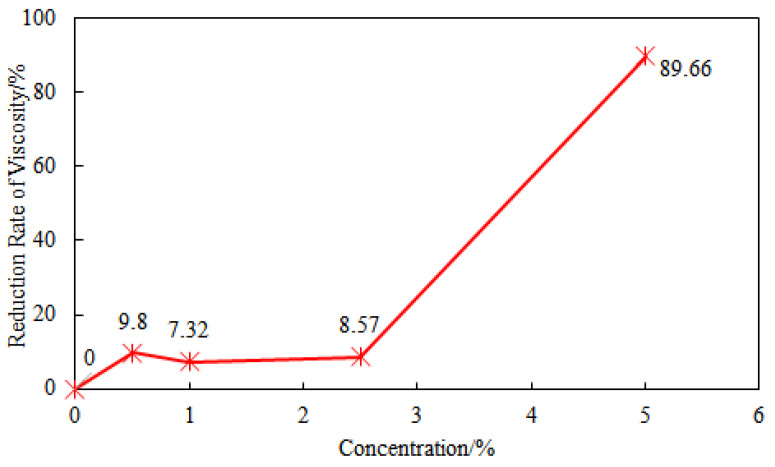
Viscosity reduction rate of the same drilling fluid before and after hot rolling.

Comparing the viscosity of the drilling fluid before and after hot rolling with a certain concentration of NaCl, it can be found that when the NaCl concentration is below 2.5%, the viscosity reduction rates before and after hot rolling are 9.8%, 7.32%, and 8.57%, showing stable performance. However, when the NaCl concentration is 5%, the viscosity reduction rate of the drilling fluid increases dramatically to 89.66% (Figure 8). This sudden change in viscosity is caused by percolation-type phase transition due to the coupling of high temperature and high salt fields, with mechanisms including chain contraction induced by electrostatic shielding [35], disentanglement of entanglement networks caused by salt-induced phase separation [36], and molecular weight cliff-like decline caused by thermal oxidative chain scission degradation [37].

### 2.7. Performance of GW-VIS in Saturated Saltwater

A 2% polymer water suspension was prepared, and sodium chloride was added to saturate it. The changes in viscosity and shear force of the water suspension after hot rolling at 180 °C, 200 °C, and 220 °C were tested.

Comparing the performance of Experiment 1 and Experiment 3, it can be seen that after adding saturated sodium chloride, the viscosity of GW-VIS water suspension decreased from 72.5 mPa·s to 52 mPa·s, a decrease of 28.27%. This indicates that the initial viscosity decreased due to charge shielding, where salt ions weakened the electrostatic repulsion of the polymer by compressing the double layer, causing the molecular chains to curl and the hydrodynamic radius to decrease. The YP/PV ratio decreased from 0.81 to 0.44, indicating that the presence of salt caused the system to transition from an elastic structure fluid (high YP) to a viscous fluid, and the electrostatic network structure collapsed [38]. After hot rolling at 180 °C, the viscosity reduction rates for the saturated saltwater system and the clear water system were 0 and 1.4%, indicating that when the polymer concentration is high (such as 2%), the hydrophobic association effect between molecules dominates. Even under saturated saltwater and high temperature (180 °C) conditions, stable supramolecular network structures can still be formed between molecules. This network structure is maintained by the association effect of hydrophobic groups and can resist the shielding effect of salt ions on charges, so the viscosity does not change significantly [39]. As shown in the data from Figure 6, when the dosage of GW-VIS is 1 wt%, the addition of 5 wt% NaCl resulted in a 49.09% reduction in viscosity of the suspension before hot rolling and a 94.55% decline after hot rolling (16 h at 180 °C). The viscosity difference between pre- and post-hot rolling under NaCl conditions was 89.66% (Figure 7). In contrast, when the GW-VIS dosage was increased to 2 wt%, the viscosity remained unchanged even under saturated NaCl before and after 180 °C hot rolling (Table 3). This behavior aligns with the Flory–Huggins solution theory discussed in Figure 5. When the polymer concentration exceeds the critical overlap concentration (C*), viscosity increases dramatically due to chain entanglement. Similarly, in saturated brine, although NaCl induces chain coiling of GW-VIS molecules through charge screening and salting-out effects, a sufficiently high polymer concentration ensures robust viscosification performance. This reduces the detrimental impact of NaCl on rheological stability, as interchain hydrophobic associations and physical entanglements dominate over salt-induced polymer collapse. Research indicates that hydrophobic association polymers will increase in viscosity after reaching the critical salinity, as the intermolecular association is enhanced. Although the high concentration of sodium chloride may have exceeded this critical value, the intermolecular association is already strong enough at high polymer concentrations, and further increasing the salinity does not destroy its structure. At 200 °C, the viscosity of the saturated saltwater system decreased significantly to 43 mPa·s (a decrease of 17.31%), and the YP/PV ratio dropped to 0.43, indicating that the molecular chains began to thermally degrade and entangle, reducing the structural viscosity. At 220 °C, the viscosity dropped sharply to 20 mPa·s (a decrease of 61.54%), and the YP/PV ratio was only 0.33, indicating that the main chain underwent breakage and degradation (such as C-O or C-C bond breakage), and Cl- in the saturated saltwater may catalyze free radical reactions, accelerating the degradation of GW-VIS.

### 2.8. Performance Comparison Between GW-VIS and HE300

A water-based tackifier suspension identical to that described in Section 2.5 was prepared and hot rolled at 180 °C for 16 h. The performance of GW-VIS was compared with HE300, a commercial product from Chevron Corporation, as shown in Table 4.

The comparison reveals that GW-VIS exhibited superior viscosifying capabilities. After hot rolling at 180 °C, GW-VIS demonstrated higher viscosity, a lower viscosity reduction rate, and a greater YP/PV ratio, indicating enhanced performance stability under high-temperature conditions.

### 2.9. Field Application Performance of GW-VIS

To validate the field application effectiveness of GW-VIS, field trials were conducted in three wells located in the Liaohe oilfield. The wells were designed to have depths exceeding 3100 m, with a geothermal gradient of 3.6 °C per 100 m. The system used was a clay-free water-based drilling fluid, with the core treatment agent being the independently developed GW-VIS. The specific drilling fluid formulation included 0.5% GW-VIS + 2% sepiolite.

During the field trial of well #1, the tackifier was directly added to the mud circulation tank from the packaging bag, stirred for 2 h, and then drilling commenced (Table 5). The initial apparent viscosity was only 13 mPa·s. As the drilling fluid circulated, the undissolved tackifier continued to dissolve, gradually increasing the viscosity of the drilling fluid. Throughout the drilling process, the clay-free drilling fluid performed well, with a stable yield ratio, indicating that the product did not undergo significant degradation and the tackifier performed excellently. The output of this well has increased by 15% compared with that of the adjacent well.

During the field trial of well #2, the tackifier was added to an empty tank, mixing with water and other additives and stirred for 2 h, then mixed into the drilling fluid (Table 6). GW-VIS was continuously circulated for 4 h. The performance of the drilling fluid did not change significantly, but the viscosity decreased slightly at the fourth hour, requiring some replenishment. The output of this well has increased by 12.4% compared with that of the adjacent well.

From the test results of well #3, the tackifier was added to an empty tank, mixing with water and other additives and stirred for 5 h, then mixed into the drilling fluid (Table 7). It can be seen that when the tackifier was pre-mixed and stirred for 5 h, its dissolution performance improved. During use, the performance was very stable, indicating that the product performed well. The output of this well has increased by 8.6% compared with that of the adjacent well.

## 3. Conclusions

The synthetic GW-VIS, with its linear molecular chains and adsorption groups (amide, sulfonic acid, etc.), exhibits excellent viscosity-enhancing properties. By forming a network structure through molecular entanglement, it effectively increases the viscosity of drilling fluids. A dosage of 0.5% is sufficient to meet the requirements of vertical well operations (k = 761). This feature enables GW-VIS to achieve the same tackifying effect as existing commercial products with very little addition. All raw materials are purchased and produced by us, which can greatly reduce costs.

GW-VIS maintains its viscosity without attenuation after hot rolling at 180 °C for 16 h, with a temperature resistance limit of up to 200 °C (reduction rate of 14.8%). When the dosage is 2%, the anti-sodium chloride contamination of GW-VIS can reach saturation. This makes GW-VIS have a very broad application prospect in high-temperature wells.

Field trials have proved that GW-VIS with a 0.5% addition can meet the drilling requirements in the clay-free drilling fluid, and the rheological stability and rock-carrying capacity of the system meet the construction requirements of drilling.

## 4. Materials and Methods

### 4.1. Materials

The main chemical materials used in the experiment include: acrylamide, AMPS, NVP, nano calcium carbonate, ammonium persulfate, sodium bisulfite, sodium hydroxide, absolute ethanol, etc., all of which are analytical grade and purchased from Macklin Reagent Network; high-purity nitrogen gas (N_2_ concentration of 99.999%), purchased from Beijing Chengwei Xin Industrial Gas Sales Company (Beijing, China).

The instruments used in the experiment include: Brookfield DV2T rotational viscometer, purchased from the Brookfield Engineering Laboratories (Middleboro, MA, USA); GLS-4 digital high-speed stirrer, purchased from Shandong Meiling Scientific Instrument Co., Ltd. (Linzi, China); constant temperature water bath, purchased from Changzhou Yaowang Instrument Co., Ltd. (Changzhou, China); three-neck flask, beaker, graduated cylinder, glass stopper, purchased from Shenlan Instrument Company (Shanghai, China); TGA-4000 thermogravimetric analyzer, purchased from PerkinElmer Company (Waltham, MA, USA); deionized water, self-made in the laboratory; Nexus Fourier infrared spectrometer, purchased from Thermo Nicolet Company (Madison, WI, USA).

### 4.2. Methods

#### 4.2.1. Preparation of the Polymer Gel Tackifier

GW-VIS was prepared by water suspension polymerization, with the following specific steps: Dissolve 3 g of AMPS in deionized water to prepare a 50% water suspension, adjust the pH of the water suspension to neutral with sodium hydroxide to obtain an AMPS(Na) water suspension. After the AMPS(Na) suspension cools to room temperature, add 1 g AM and 0.05 g nano calcium carbonate into the suspension, introduce high-purity nitrogen gas, and stir for 30 min to remove oxygen. Add initiator at 0.1% of the monomer weight (ammonium persulfate and sodium bisulfite mixed in a 1:1 ratio), stir at 35 °C for 1 h. Add 1 g NVP and 0.006 g crosslinking agent MBA, introduce nitrogen for 30 min, then add a certain amount of initiator, raise the temperature to 45 °C, and react for 5 h to obtain a highly viscous gel-like product. Cut the product into pieces and add it to deionized water, stirring continuously until swelling and even dissuspension. Add acetone to precipitate the reaction product and wash three times, dry in vacuum at 45 °C to obtain the polymer gel tackifier named GW-VIS.

#### 4.2.2. Performance of GW-VIS

- Infrared Spectroscopy of GW-VIS

Use the Nexus Fourier infrared spectrometer to test the infrared spectrum curve of GW-VIS. Take a small amount of cleaned and purified product, add it to an agate mortar, mix it with a small amount of analytical pure KBr, grind it thoroughly to mix evenly, place the mixture in a sample screw, tighten it with a wrench, open the screw, and if the sample appears as a transparent thin slice, the sample preparation is successful. Place the sample on the sample stage for testing [40].

- Scanning Electron Microscopy Analysis of GW-VIS

Use the ZEISS sigma300 scanning electron microscope to observe the microscopic morphology of the polymer gel tackifier. Take a small amount of cleaned, purified, and dried product, evenly attach it to the conductive adhesive, ensure good contact between the sample and the conductive adhesive, and spray gold on the surface to improve conductivity [41]. Place the conductive adhesive with the attached sample in the scanning electron microscope sample chamber to observe the microscopic morphology of the gel.

- Viscosity of GW-VIS water suspension

Add 4 g of GW-VIS powder to 400 mL of deionized water, stir at 10,000 r/min for 30 min, and use a six-speed rotational viscometer and Brookfield viscometer to test the viscosity of the GW-VIS water suspension. The method for testing viscosity with the six-speed rotational viscometer is as follows. Pour the sample to be tested into the sample cup and place it on the sample cup holder of the instrument. Adjust the height so that the liquid level is exactly at the measurement line of the drum. Adjust the viscometer speed to 600 r/min, wait for the reading to stabilize, read and record the value. Use the same method to test the value at 300 r/min [42]. Calculate the apparent viscosity (AV) and the n- and K-values of the drilling fluid according to the following formulas:

AV = Ф600/2,(1)

*n* = 3.322 × lg(Ф600/Ф300), (2)

*K* = 511 × Ф600÷1022^n^, (3)

- Thermal Resistance Performance of GW-VIS water suspension

Add 4 g of GW-VIS powder and 0.5% high-temperature stabilizer to 400 mL of deionized water, stir at 10,000 r/min for 30 min, and use a six-speed rotational viscometer to test the viscosity of the GW-VIS water suspension. Pour the gel water suspension into a high-temperature aging tank, heat roll at 180 °C, 200 °C, and 220 °C for 16 h, test the viscosity of the water suspension at room temperature after hot rolling, and calculate the viscosity reduction rate.

- Rheological Performance of GW-VIS in Saltwater suspension

Add a certain amount of GW-VIS powder to 400 mL of deionized water, dissolve it, add different concentrations of NaCl, stir at 10,000 r/min for 30 min, and use a six-speed rotational viscometer to test the viscosity of the GW-VIS saltwater suspension. Pour the GW-VIS saltwater suspension into a high-temperature aging tank, heat roll at 180 °C for 16 h, and test the viscosity of the saltwater suspension at room temperature after hot rolling. Place the water suspension in the sample cup and place it on the sample cup holder of the instrument. Adjust the height so that the liquid level is exactly at the measurement line of the drum. Adjust the viscometer speed to 600 r/min, wait for the reading to stabilize, read and record the value. Use the same method to test the values at 300 r/min, 200 r/min, 100 r/min, 6 r/min, and 3 r/min. Calculate the plastic viscosity (PV) and yield point (YP) of the drilling fluid according to the following formulas:

PV = Ф600/2 − Ф300,(4)

YP = AV − PV,(5)

- Thermal Resistance Performance of GW-VIS in Saturated Saltwater Suspension

Add 2% of GW-VIS powder to 400 mL of deionized water, dissolve it, add 30% of NaCl, stir at 10,000 r/min for 30 min, and use a six-speed rotational viscometer to test the viscosity of the GW-VIS saltwater suspension. Pour the gel saltwater suspension into a high-temperature aging tank, heat roll at 180 °C, 200 °C, and 220 °C for 16 h, and test the viscosity of the saltwater suspension at room temperature after hot rolling using a six-speed rotational viscometer.

## Figures and Tables

**Table 1:** *K* and *n* of gel water suspensions at different concentrations.

Concentration/%	0.05	0.1	0.25	0.5	0.75	1
*n*	1	0.68	0.71	0.49	0.42	0.43
*K*	1	19	67	761	2066	2863

**Table 2:** Rheological properties of GW-VIS water suspensions.

Temperature	Condition	AV/(mPa·s)	PV/(mPa·s)	YP/Pa	YP/PV	Viscosity Reduction Rate/%
	Before hot rolling	27.5	17	10.5	0.62	0
180	After hot rolling	27.5	18	9.5	0.53	0
200	After hot rolling	23	15	8	0.53	14.80
220	After hot rolling	13.5	10	3.5	0.35	50.90

**Table 3:** Rheological properties of GW-VIS suspension.

No.	Temperature/°C	Condition	AV/(mPa·s)	PV/(mPa·s)	YP/Pa	YP/PV	Viscosity Reduction Rate/%
1	25 (Water)	Before hot rolling	72.5	40	32.5	0.81	/
2	180 (Water)	After hot rolling	71.5	40	31.5	0.79	1.4
3	25 (Saturated saltwater)	Before hot rolling	52	36	16	0.44	/
4	180 (Saturated saltwater)	After hot rolling	52	35	17	0.49	0
5	200 (Saturated saltwater)	After hot rolling	43	30	13	0.43	17.31
6	220 (Saturated saltwater)	After hot rolling	20	15	5	0.33	61.54

**Table 4:** Comparison of rheological properties of viscosifier solutions (GW-VIS and HE300).

Tackifier	Condition	AV/(mPa·s)	PV/(mPa·s)	YP/Pa	YP/PV	Viscosity Reduction Rate/%
GW-VIS	Before hot rolling	27.5	17	10.5	0.62	0
	After hot rolling	27.5	18	9.5	0.53	0
HE300	Before hot rolling	20.5	14	6.5	0.46	0
	After hot rolling	19.5	13	6.5	0.50	4.88

**Table 5:** Field trial data for well #1.

Well Depth/m	AV/(mPa·s)	PV/(mPa·s)	YP/Pa	YP/PV	φ6/φ3	FL_API_/mL	n	K
3658	13	9	4	0.44	2/1	14	0.61	190
3715	13.5	10	3.5	0.35	2/1	9.8	0.67	137
3771	17	12	5	0.42	2/1	9.6	0.63	224
3803	21	15	6	0.40	2/1	9.7	0.64	259
3854	22.5	16	6.5	0.41	2/1	9.6	0.63	285

**Table 6:** Field trial data for well #2.

Time Added/h	AV/(mPa·s)	PV/(mPa·s)	YP/Pa	YP/PV	φ6/φ3	FL_API_/mL	n	K
1	34.5	24	10.5	0.44	6/5	7.6	0.62	477
2	30	21	9	0.43	5/4	7.4	0.62	413
3	28.5	20	8.5	0.43	5/4	7.6	0.62	387
4	22	16	6	0.38	4/3	8.2	0.65	245

**Table 7:** Field trial data for well #3.

Well Depth/m	Time Added/h	AV/(mPa·s)	PV/(mPa·s)	YP/Pa	YP/PV	φ6/φ3	FL_API_/mL	n	K
3116	0	16.5	11	5.5	0.5	2/1	8.8	0.58	293
3126	2	17.5	12	5.5	0.46	2/1	9.0	0.61	269
3165	13	16.5	11	5.5	0.5	2/1	8.6	0.58	293
3178	18.5	16	11	5	0.45	2/1	8.6	0.61	243
3193	23	17	12	5	0.42	2/1	8.4	0.63	224
3193	25.5	17.5	12	5.5	0.46	φ2/13	8.4	0.61	269

## Data Availability

The original contributions presented in this study are included in the article. Further inquiries can be directed to the corresponding author.
